# How radiation influences atherosclerotic plaque development: a biophysical approach in ApoE ¯/¯ mice

**DOI:** 10.1007/s00411-017-0709-2

**Published:** 2017-09-02

**Authors:** Astrid Kloosterman, Teun van Dillen, Harmen Bijwaard, Sylvia Heeneman, Saske Hoving, Fiona A. Stewart, Fieke Dekkers

**Affiliations:** 1 Centre for Environmental Safety and Security, National Institute for Public Health and the Environment (RIVM), Bilthoven, The Netherlands; 2 Medical Technology Research Group, Inholland University of Applied Sciences, Haarlem, The Netherlands; 3 Experimental Vascular Pathology group, Department of Pathology, Cardiovascular Research Institute Maastricht (CARIM), Maastricht University Medical Center, Maastricht, The Netherlands; 4 Division of Biological Stress Response (H3), Netherlands Cancer Institute - Antoni van Leeuwenhoek Hospital, Amsterdam, The Netherlands

**Keywords:** Atherosclerosis, Mathematical modeling, ApoE$^{{-/-}}$ mice, Ionizing radiation

## Abstract

Atherosclerosis is the development of lipid-laden plaques in arteries and is nowadays considered as an inflammatory disease. It has been shown that high doses of ionizing radiation, as used in radiotherapy, can increase the risk of development or progression of atherosclerosis. To elucidate the effects of radiation on atherosclerosis, we propose a mathematical model to describe radiation-promoted plaque development. This model distinguishes itself from other models by combining plaque initiation and plaque growth, and by incorporating information from biological experiments. It is based on two consecutive processes: a probabilistic dose-dependent plaque initiation process, followed by deterministic plaque growth. As a proof of principle, experimental plaque size data from carotid arteries from irradiated ApoE$^{{-/-}}$ mice was used to illustrate how this model can provide insight into the underlying biological processes. This analysis supports the promoting role for radiation in plaque initiation, but the model can easily be extended to include dose-related effects on plaque growth if available experimental data would point in that direction. Moreover, the model could assist in designing future biological experiments on this research topic. Additional biological data such as plaque size data from chronically-irradiated mice or experimental data sets with a larger variety in biological parameters can help to further unravel the influence of radiation on plaque development. To the authors’ knowledge, this is the first biophysical model that combines probabilistic and mechanistic modeling which uses experimental data to investigate the influence of radiation on plaque development.

**Electronic supplementary material:**

The online version of this article (doi:10.1007/s00411-017-0709-2) contains supplementary material, which is available to authorized users.

## Introduction

It is well known that cardiovascular diseases may occur as a side effect of radiotherapy (Stewart et al. 2013). In recent years, evidence has emerged indicating that exposure to ionizing radiation may have detrimental effects on the circulatory system (Schultz-Hector and Trott 2007; Hendry et al. 2008; Darby et al. 2010), including an extensive Life Span Study based on a cohort of survivors of the atomic bombs in Hiroshima and Nagasaki where the whole body was exposed to ionizing radiation (Shimizu et al. 2010). There is strong evidence that the origin of such circulatory diseases lies in a dysfunction of the endothelial cells (i.e., inner lining of a blood vessel). This sets off a chronic inflammatory response serving as the origin of atherosclerotic plaque development (Lusis 2010). However, its exact underlying biological mechanism and how ionizing radiation may increase or promote such vascular tissue damage is not exactly known (Schultz-Hector and Trott 2007; Stewart et al. 2010; Khaled et al. 2012). In this article we shed light on the origin of atherosclerotic plaque development and its possible stimulation by radiation exposure. This work can help to explore the possible effects to the circulatory system in the low-dose regime, which is of importance to radiation protection (up to several tenths of Grays), since the nature of these effects is still under debate (Hildebrandt 2010; Gabriels et al. 2014; Mancuso et al. 2015; Mitchel et al. 2011).

Our approach is based on a sophisticated biomathematical model of atherosclerotic plaque growth developed by Ougrinovskaia et al. (2010). This model incorporates several crucial aspects of the chronic inflammatory process as a response of the immune system to cholesterol in the arterial wall after invasion through the endothelial-cell barrier. We extended the model to include the probabilistic nature of individual plaque initiation and effects on plaque initiation induced by exposure to ionizing radiation, and made it suitable for incorporating experimental data. To illustrate this, the model was tailored to experimental plaque size data from irradiated ApoE$^{{-/-}}$ mice, which are prone to develop atherosclerosis. The results show how the model can provide insight into the underlying biological processes of plaque development including possible radiation effects. The reader should realize that the focus of this manuscript is on the developed methodology, rather than on the exact quantitative results.

## Construction of the model

### Experimental data and descriptive analysis

**Fig. 1:**
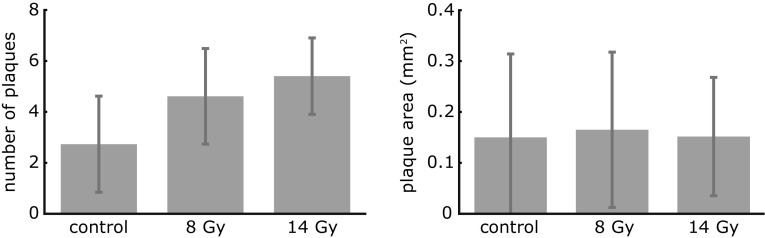
*Bar graphs* show the average numbers of plaques per mouse (*left*) and average plaque areas (*right*) for the three dose groups [*0* (*control*), *8*, and *14 Gy*]. The *error bars* indicate the corresponding standard deviations. From these results, a dose-related effect would be more likely to be present in the plaque initiation process than in the volumetric plaque growth

Experimental plaque size data was available from carotid arteries from locally irradiated ApoE$^{{-/-}}$ mice with 8 and 14 Gy and from a sham-treated control group (0 Gy) (Hoving et al. 2008). The total study consisted of 30 female ApoE$^{{-/-}}$ mice with 8 to 12 mice per dose group. All mice were fed a standardized mouse chow diet (3.7 % fat). X-ray irradiation took place at the age of 13–14 weeks, and all mice were sacrificed at the age of 42–44 weeks for plaque size measurements. These consisted of measuring longitudinal cross-sectional areas of the plaques in the carotid arteries. In most cases, a mouse had multiple plaques. For all mice, the size of each individual plaque was represented by one measured plaque area. For each dose group [0 (control), 8, and 14 Gy], the number of plaques per mouse and the measured areas were averaged over the mice in the dose group. Figure 1 shows these averages and the corresponding standard deviations. Although not statistically significant, the number of plaques per mouse seems to increase with radiation dose, while the effect of radiation dose on plaque area is not convincing from these data. This suggests that radiation, as delivered in this study, is more likely to act on plaque initiation than on the volumetric plaque growth of a plaque after it has been initiated. This motivated us to consider plaque development as two consecutive processes: plaque initiation and plaque growth. Plaque initiation was modeled as a probabilistic dose-dependent process and plaque growth was described by a mechanistic model independent of the delivered dose, and thus identical for all mice. Both processes will be discussed separately in the next two Sections. Note that in this model, plaque growth means volumetric growth of an individual plaque and not the increase of the number of plaques or the increase of the total plaque volume of all plaques in the carotid artery.

### Plaque initiation

The observed size of a plaque does not only depend on the features of the growth process, which will be discussed in the next Section, but also on the age or time at which the plaque is created: the so-called initiation time. Plaques formed earlier in time have had a longer period to grow and are therefore larger in size under the assumption that the growth process is identical for all plaques. This initiation process can be related to dysfunction of the endothelial cells and may be affected by exposure to ionizing radiation. This is the start of the subsequent chronic inflammatory response. In the current model, the nature of the initiation process was assumed to be probabilistic, but any spatial dependency (i.e., location of plaque in the blood vessel) was not regarded. Initiation events were assumed to follow a non-homogeneous Poisson process with an event rate $\lambda$. In this work, $\lambda$ is the expected average number of initiations per unit time. The event rate may depend on several parameters denoted by vector $\varvec{\theta }$ and on age *t*: $\lambda = \lambda \left( t, \varvec{\theta } \right)$. The integrated event rate, $\Lambda _{t_{1},t_{2}} \left( \varvec{\theta } \right)$, is the average number of plaques initiated between age $t_{1}$ and $t_{2}$, and is defined by:

1 $$\begin{aligned} \Lambda _{t_{1},t_{2}} \left( \varvec{\theta } \right) = \int _{t_1}^{t_{2}} \lambda \left( t, \varvec{\theta } \right) \, \mathrm{d}t. \end{aligned}$$

**Fig. 2:**
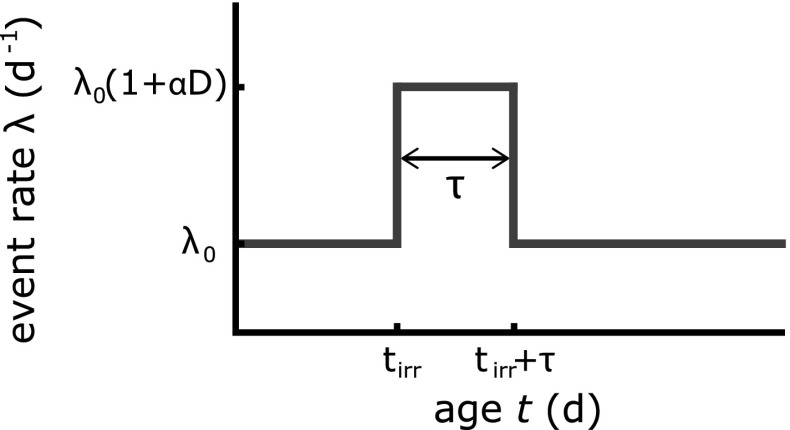
The event rate used to model the plaque initiation process represents the expected average number of plaque initiations per day and consists of a baseline $\lambda _{0}$ which is elevated for some time $\tau$ after irradiation at age $t_\mathrm{irr}$. The event rate during this period is modeled to depend on the dose *D*

In the absence of radiation, the baseline event rate was modeled to be constant at a value of $\lambda _{0}$ for all mice. Exposure to ionizing radiation was regarded as an effect modifier of the baseline event rate. For an irradiated mouse, the event rate was assumed to be elevated during some time $\tau$ after irradiation at age $t_\mathrm{irr}$. After this period, we assumed the event rate to return to its baseline value of $\lambda _{0}$. The level of elevation was modeled to depend linearly on the delivered dose *D* (in Gy), and consequently the event rate was finally written as

2 $$\begin{aligned} \lambda \left( t, \varvec{\theta } \right) = \lambda _{0} \left( 1+\alpha D \left[ H \left( t-t_\mathrm{irr} \right) - H \left( t-t_\mathrm{irr}-\tau \right) \right] \right) \end{aligned}$$

with $\varvec{\theta }=\left( \lambda _{0}, \alpha \right)$ and *H*(*t*) the Heaviside step function ($H(t)=0$ for $t<0$ and $H(t)=1$ for $t\ge 0$). Figure 2 shows the functional behavior of Eq. (2). The value for time period $\tau$ was set equal to 14 days (Tribble et al. 1999).

### Plaque growth

Volumetric growth of a plaque after initiation can be described by a mechanistic model developed by Ougrinovskaia et al. (2010). A set of rate equations for the concentrations of modified LDL particles *l*(*s*), the monocyte-derived macrophage capacity concentration (i.e., the vacancy concentration for modified LDL particles in macrophages) *m*(*s*), and the internalized lipid content *n*(*s*) describes the early stages of plaque growth as a function of growth time *s*, with $s=0$ at time of initiation. Taking the uptake parameter equal for the three equations for continuity, this set of equations reads:

3 $$\begin{aligned} \frac{\mathrm{d}l}{\mathrm{d}s}&= F_{0} - \rho _{1} U\left( l \right) m \end{aligned}$$

4 $$\begin{aligned} \frac{\mathrm{d}m}{\mathrm{d}s}&= F_{m}l + \rho _{2} R \left( l, m \right) l m - \rho _{1} U\left( l \right) m \end{aligned}$$

5 $$\begin{aligned} \frac{\mathrm{d}n}{\mathrm{d}s}&= \rho _{1} U\left( l \right) m \end{aligned}$$

where $U(l)=\frac{l}{1+l/l_\mathrm{th}}$ is the uptake function, which limits the ingestion rate of modified LDL particles by macrophages to $l_\mathrm{th}$, and $\rho _{1}$ is the uptake parameter. In Eq. (3), $F_{0}$ is the concentration modified LDL particles entering the intima per unit time and is assumed to be constant over time. Further, $F_{m}$ in Eq. (4) is the capacity concentration that is recruited due to the presence of modified LDL per unit time and per unit of modified LDL concentration in the intima. Furthermore, Eq. (4) comprises another influx term regulated by cytokines released by both macrophages and T-cells. Down-regulation by T-cells is described by a response function $R(l,m) = \frac{1}{1+ \epsilon {\left( m/l \right) }^2 }$ depending on the ratio *m* / *l*, with a feedback constant $\epsilon$. The parameter $\rho _{2}$ is the response parameter. Equations (3)–(5) are similar to those in Ougrinovskaia et al. (2010), but with each uptake parameter equal to $\rho _{1}$. Since analysis of the observed plaque size data with descriptive statistics did not show a convincing dependence on radiation dose, the mechanistic model was not adapted to include influences of radiation action. Finally, we assumed for simplicity that plaque volume *V* is proportional to the internalized lipid concentration: $V \left( s \right) = \kappa \cdot n \left( s \right)$, with $\kappa$ the constant of proportionality. We refer to Ougrinovskaia et al. (2010) for a more detailed description of the mechanistic model. We emphasize that in this first approach to model plaque size data, the mechanistic model was treated as a deterministic process: each plaque has a similar growth curve after its initiation.

### Numerical analysis: combining initiation and growth

The plaque development model contains several parameters: the parameters describing the event rate $\lambda$ in the Poisson process that models plaque initiation and the parameters in the set of differential equations that models plaque growth. For some parameter values, in this study $F_{0}$, $l_\mathrm{th}$, and $\epsilon$, reasonable estimates could be made from literature (see next section and Table 1). These parameters were defined as fixed parameters and were fixed to these estimated values. The remaining parameters were defined as fit parameters or also called free parameters. In this case the free parameters were described by $\{ \varvec{\theta }, \varvec{\beta } \}$, with $\varvec{\theta } = ( \lambda _{0},\alpha )$ from the probabilistic initiation model, and $\varvec{\beta } = ( \rho _{1},\rho _{2},F_{m}, \kappa )$ from the mechanistic growth model. By combining the probabilistic plaque initiation model and the mechanistic growth model, we constructed a likelihood function that enabled a fitting procedure of the experimental plaque size data from the ApoE$^{{-/-}}$ mice. A maximization of this likelihood function then yielded optimal values $\{ \varvec{\hat{\theta }}, \varvec{\hat{\beta }} \}$ of the model’s free parameters. The followed procedure is explained hereafter.

**Table 1:** Numerical values for the fixed parameters, free parameters, and the deviances resulting from the optimizations are listed for two typical plaque development scenarios

	Fixed parameters				Free parameters						Dev
	$F_{0}$	$l_\mathrm{th}$	$\epsilon$	$\tau$	$\rho _{1}$	$\rho _{2}$	$F_{m}$	$\kappa$	$\lambda _{0}$	$\alpha$	
	[M $\cdot$ s$^{-1}$]	[M]	[-]	[d]	[M$^{-1}\cdot$d$^{-1}$]	[M$^{-1}\cdot$d$^{-1}$]	[s$^{-1}$]	[l $\cdot$ M$^{-1}$]	[d$^{-1}$]	[Gy$^{-1}$]	
Scenario 1	$4 \cdot 10^{-13}$	$3 \cdot 10^{-4}$	$10^{-10}$	14	$0.8 \cdot 10^{-3}$	$5.2 \cdot 10^{3}$	$2.5 \cdot 10^{-3}$	0.48	0.013	0.17	1313
Scenario 2	$4 \cdot 10^{-13}$	$3 \cdot 10^{-4}$	$10^{-5}$	14	$0.3 \cdot 10^{-3}$	1.9	$5.2 \cdot 10^{-3}$	4.0	0.013	0.07	1320

We started by considering all *N* mice in a certain dose group [0 (control), 8, and 14 Gy] and constructed the individual likelihood for mouse *j* which had obtained $n_{j}$ plaques at attained age $T_{j}$. For mouse *j*, the experimentally obtained plaque volumes were $\tilde{V}_{i,n_{j}}^{j}$ (plaque index $i=1,2,\ldots ,n_{j}$ ), where the tilde refers to experimental values. For a certain set of free model parameters $\left\{ \varvec{\theta }, \varvec{\beta } \right\}$, the mechanistic model described the corresponding plaque growth curve $V \left( s, \varvec{\beta } \right)$. From this we could reconstruct the growth time $s_{i,j} \left( \varvec{\beta } \right)$ of each plaque *i* by setting $V \left( s_{i,j} \left( \varvec{\beta } \right) , \varvec{\beta } \right) = \tilde{V}_{i,n_{j}}^{j}$ , and the corresponding initiation time as $t_{i,j}\left( \varvec{\beta } \right) = T_{j}-s_{i,j}\left( \varvec{\beta } \right)$. The time step we used was one day. These initiation times were ordered such that $0 \le t_{1,j}<t_{2,j}< \cdots<t_{n_{j},j}<T_{j}$. The individual likelihood function then followed from the joint probability of the set of initiation times $t_{i,j} \left( \varvec{\beta } \right)$ of the $n_{j}$ plaques based on the non-homogeneous Poisson process with event rate $\lambda _{j} \left( t,\varvec{\theta } \right)$ for mouse *j* (Eq. (2)). Next, all individual likelihoods among the *N* mice were multiplied, resulting in the dose group’s total likelihood function (Cox and Lewis 1966; Lawless 1987):

6 $$\begin{aligned} \mathcal {L} \left( \varvec{\theta }, \varvec{\beta } \right) = \prod _{j=1}^{N} \left\{ \prod _{i=1}^{n_{j}} \lambda \left( t_{i,j} \left( \varvec{\beta } \right) , \varvec{\theta } \right) \right\} \exp \left[ -\Lambda _{0,T_j}^{j} \left( \varvec{\theta } \right) \right] \end{aligned}$$

with $\Lambda _{0,T_j}^{j}$ the lifetime-integrated event rate for mouse *j* using Eq. (1). Finally, the grand total likelihood was calculated by multiplying the likelihood functions of all dose groups. Using the optimization routine of adaptive simulated annealing (ASA) (Ingber 1993, 2004), the model’s free parameters $\left\{ \varvec{\theta }, \varvec{\beta } \right\}$ were varied until a maximum of the likelihood was found. Maximization of the likelihood was in fact accomplished by minimizing the deviance value Dev$\left( \varvec{\theta }, \varvec{\beta } \right) =-2 \ln \mathcal {L}\left( \varvec{\theta }, \varvec{\beta } \right)$, with $\left\{ \varvec{\hat{\theta }}, \varvec{\hat{\beta }} \right\} = \underset{\varvec{\theta }, \varvec{\beta }}{\arg \min}\,\mathrm{Dev} \left( \varvec{\theta }, \varvec{\beta } \right)$ the optimal set of parameters. The optimal parameter search was constrained by a range of reasonable values obtained from literature, which will be discussed in the next Section.

To apply the model to experimental data, the quantities *l*, *m*, and *n* in Eqs. (3)–(5) were expressed as molar concentrations M (mol/l). Since the experimental plaque size data consisted of longitudinal cross-sectional plaque areas $\tilde{A}$, the volumes of individual plaques were estimated as $\tilde{V}=\tilde{A}^{3/2}$ under the assumption that volumetric plaque growth is similar in all three spatial dimensions. This means that plaques become thicker and will cover a larger part of the blood vessel as well during plaque progression.

## Experimental parameter values from literature

We used experimental parameter values from literature to make estimates for the fixed parameters to illustrate how known parameter values were incorporated in the model. The influx parameter $F_{0}$ in Eq. (3) was defined as an increase in concentration of modified LDL in the intima. This increase is a consequence of both the influx of LDL into the intima and LDL oxidation in the intima. The study described in Cobbold et al. (2002) suggests an LDL influx into the intima of $3.84 \cdot 10^{-5} \upmu\mathrm{M/s}$. LDL oxidation occurs on a time scale of hours to days, which can be considered small compared to the time scale of plaque formation (Cobbold et al. 2002) (which is in our case in the order of 100 days on average). Since variations in LDL plasma concentrations are assumed to be insignificant without significant changes in diet, the influx of modified LDL particles was expressed as a constant influx. However, a constant increase in concentration of modified LDL in the intima of $3.84 \cdot 10^{-5} \upmu\mathrm{M/s}$ implies a solution of pure LDL only 90 days after the increase started, assuming a spherical LDL particle with a radius of 11 nm (Yang and Vafai 2006). To use a more realistic influx, we chose to reduce the value by a factor of 100. This choice of reduction factor is somewhat arbitrary, but it can be a good starting point to illustrate the applicability of the model using experimental plaque size data. Further, the threshold parameter $l_\mathrm{th}$ in the uptake function was taken equal to the concentration of pure LDL, which is 0.3 mM.

The first parameter related to the influx of the monocyte-derived macrophage capacity appears in Eq. (4) as $F_{m}$. From in vitro experiments with Human Umbilical Vein Endothelial Cells (HUVEC) described in Shang and Issekutz (1998), the spontaneous influx of monocytes (i.e., in the absence of modified LDL) can be estimated by $1.3 \cdot 10^{-20}$ M/s. The study in Cushing et al. (1990) suggest a 2 to 3 times increased influx in monocytes with the presence of 20 $\upmu$g/ml minimally modified LDL (Heinecke et al. 1991), which corresponds with a concentration of 7 nM. Combining these two values resulted in a monocyte influx equal to $2.9 \cdot 10^{-12}$/s, since this monocyte influx was modeled to be proportional to the concentration of modified LDL. However, the value for $F_{m}$ is related to the capacity influx, and not to the monocyte-derived macrophage influx. If we consider a foam cell as a macrophage having ingested the maximum number of modified LDL, the value for $F_{m}$ could be obtained by multiplying the estimated monocyte influx of $2.9 \cdot 10^{-12}$/s by the volume ratio of a foam cell and a modified LDL particle. This volume ratio was estimated to be ${\sim }10^{10}$ (Yang and Vafai 2006; Gerrity 1981), which yielded a value of ${\sim }10^{-2}$/s for $F_{m}$. Since a foam cell does not only consists of material originating from modified LDL particles, this value is more likely to be an upper limit. To conclude, the upper limit for the capacity influx parameter $F_{m}$ could be estimated to be $\sim 10^{-2}$/s.

The response function $R(l,m) = \frac{1}{1+\epsilon {\left( m/l \right) }^2 }$ (with feedback constant $\epsilon > 0$) in Eq. (4) models the cytokine response in the cytokine-related capacity influx. For $m \gg l$ (relatively low macrophage activity), the response function approaches 0, and for $m \ll l$ (relatively high macrophage activity), the response function approaches 1. The feedback constant $\epsilon$ defines the degree of down-regulation related to the ratio of the capacity concentration *m* and modified LDL concentration *l*. In the two typical example scenarios described in the Results Section, the values for $\epsilon$ were chosen equal to $10^{-10}$ and $10^{-5}$, such that $\epsilon {\left( m/l \right) }^2 \sim 1$ and $\epsilon {\left( m/l \right) }^2 \sim 10^{5}$ respectively.

## Visualization of the goodness-of-fit

After a model fit resulted in an optimal parameter set $\left\{ \varvec{\hat{\theta }, \hat{\beta }} \right\}$, the goodness-of-fit was visualized by comparing observed (measured) and expected (modeled) average plaque volumes. Individual plaque volumes could not be compared directly with expected modeled ones, since they can be seen as specific outcomes of a random drawing process. Therefore, we study the sorted plaque volumes: plaque volumes after sorting them according to initiation after birth. Since plaque growth is assumed to be identical for all plaques in our study, the firstly-initiated plaque of a mouse will therefore also be the largest one amongst all plaques this mouse has. To compare observed and expected plaque volumes we analyzed the average volume of the $q\mathrm{th}$ plaque in a certain dose group with *N* subjects. The observed average volume of the $q\mathrm{th}$ plaque could be determined from the experimental data by averaging the observed $q\mathrm{th}$ plaque volumes of all mice having a $q\mathrm{th}$ plaque in a dose group: $\langle V_{q}^\mathrm{obs} \rangle = \frac{1}{N_q} \sum _{j=1}^{N} \tilde{V}_{q,n_{j}}^{j}$, where $N_{q}$ is the number of mice having a $q\mathrm{th}$ plaque in the dose group (note that $\tilde{V}_{q,n_{j}}^{j}=0$ if mouse *j* does not have a $q\mathrm{th}$ plaque, i.e., for $q>n_{j}$). Next, these observed average volumes were compared with those estimated from the probabilistic biophysical model: $\langle V_{q}^\mathrm{mod} \rangle$. This quantity was computed by averaging the expected $q\mathrm{th}$ plaque volumes of all *N* mice in a dose group. It is derived in the supplementary material and reads:

7 $$\begin{aligned} \langle V_{q}^\mathrm{mod} \rangle= & {} \frac{1}{N} \sum _{j=1}^{N} \hat{V}_{q}^{j} = \frac{1}{N} \sum _{j=1}^{N} q \left( \hat{Z}_q^j \right) ^{-1} \sum _{k=q}^{\infty } \frac{1}{k!} \left( {\begin{array}{c}k\\ q\end{array}}\right) \nonumber \\&\times \int _0^{T_j} \hat{\lambda }_j \left( t \right) \left( \hat{\Lambda }_{0,t}^{j} \right) ^{q-1} \left( \hat{\Lambda }_{t,T_{j}}^{j} \right) ^{k-q} \hat{V}_j \left( T_j - t \right) \mathrm{d}t, \end{aligned}$$

with $\hat{Z}_q^j=\sum _{r=q}^{\infty } \frac{\left( \hat{\Lambda }_{0,T_j}^j \right) ^r}{r!}$, and with $\hat{\lambda }$, $\hat{\Lambda }$, and $\hat{V}$ evaluated using the optimal parameters. The large sample average in Eq. (7) accounts for all possible initiation times, which is reflected by the integral. Furthermore, it accounts for the fact that each subject *j* has a possibility of developing a total of $k \ge q$ plaques under the assumed Poisson process instead of the observed number of $n_j$ plaques. This is reflected by the summation over index *k* in combination with the normalization constant $\hat{Z}_q^j$. An average over all possible exposure and life histories is reflected by the summation over index *j*. To indicate the range of the sorted plaque volumes, the sample standard deviation was computed for both the observed as well as for the expected sorted plaque volumes.

## Results

The proposed model to describe plaque development was combined with experimental plaque size data from ApoE$^{-/-}$ mice. Different sets of initial parameter values with search intervals for the free parameters and different feedback constants $\epsilon$ were used, which resulted in different plaque initiation characteristics and plaque growth behavior. Due to the limited size of the data set, the model can describe multiple scenarios with comparable likelihood (Eq. (6)) instead of providing one convincing unique solution. Two typical scenarios are shown to illustrate the applicability of the model as a proof of concept. This does not imply that other scenarios are ruled out. The scenarios describe two typical plaque growth processes resulting from the chosen initial free parameter values and search intervals and feedback constant $\epsilon$, which were different for both scenarios. Table 1 shows the values for the fixed and free parameters and the corresponding deviances for both scenarios. The two scenarios are further discussed in the next paragraphs.

### Modeled plaque initiation

The baseline event rates are comparable for the two typical scenarios: 0.013/d. However, in the 14 days following irradiation, the elevation of the event rate is higher for scenario 1 compared to the elevation for scenario 2: $\alpha$ is 2.6 times larger in scenario 1 than in scenario 2. The averages and standard deviations of the percentage of plaques that were initiated within 14 days following irradiation per mouse are listed in Table 2 for the three dose groups. The numbers of plaques initiated in this period suggest an increase with increasing dose. This suggests that radiation acts on plaque initiation, although this effect is not statistically significant.

**Table 2:** For two typical scenarios, the ratios of the number of plaques initiated within $\tau =14$ days after irradiation (or sham irradiation) and the total number of plaques initiated during lifetime were determined for each mouse. The values represent the averages ± standard deviations of these ratios expressed as percentages per dose group

	Control	8 Gy	14 Gy
Scenario 1	0 ± 0	7 ± 13	16 ± 16
Scenario 2	5 ± 15	3 ± 7	14 ± 17

### Modeled plaque growth

**Fig. 3:**
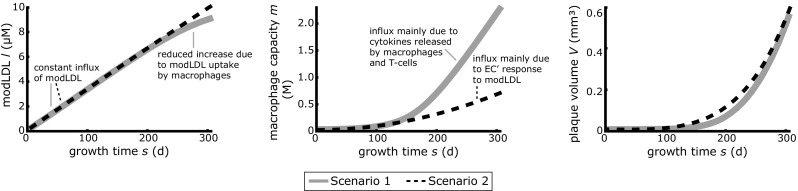
The concentrations modified LDL *l*, monocyte-derived macrophage capacity *m* as well as the plaque volumes *V* are shown as function of growth time *s* for the maximum possible growth time for the general plaque growths corresponding to scenario 1 (*gray solid lines*) and scenario 2 (*black dashed lines*). Note that plaques are modeled to be initiated at $s=0$, followed by volumetric growth. Since plaque growth is assumed to be identical for all plaques, the growth time *s* will be smaller than a mouse’s age *t*

Figure 3 shows from left to right the concentrations modified LDL *l*, the concentrations monocyte-derived macrophage capacities *m*, as well as the growth curves (plaque volume *V*) as function of growth time *s* for the two scenarios. Both scenarios show similar growth behavior, which is strongly deviating from linear growth. The constant modified LDL influx $F_{0}$ dominates the modified LDL concentration in the intima for approximately the first 250 days of growth for both scenarios. In scenario 1, the increase in concentration is lowered after these 250 days due to the increasing uptake of modified LDL by macrophages, whereas this has a minor effect in scenario 2. For both scenarios, the ingestion by macrophages is highly inefficient for the total plaque growth time. This also explains the almost linear growth of modified LDL.

For both scenarios, the macrophage capacity keeps increasing since the influx of newly-recruited macrophages into the intima on the macrophage capacity is significantly larger than the decreasing effect resulting from the uptake of modified LDL. However, the two scenarios differ in the dominant mechanism behind the macrophage influx. The total influx was modeled by an influx due to the response of the endothelial cells to modified LDL (first influx term in Eq. (4): $F_{m}l$), and an influx due to cytokines released by macrophages and T-cells as response on the macrophage activity (second influx term in Eq. (4): $\rho _{2} R \left( l, m \right) l m$). Analysis of the individual contributions of these two influxes (results not shown) yields a dominant role for the influx due to the response of the endothelial cells to the modified LDL (ratio ${\sim }10^{4}$) for scenario 2 for the total maximum growth time. Moreover, both influxes increase almost linearly with growth time in this scenario. For scenario 1 the opposite holds: the influx due to the cytokines released by macrophages and T-cells as response on the macrophage activity dominates (difference ${\sim }10^{1}$). This dominant influx as function of growth time can be considered as a sigmoid curve. The influx due to the response of the endothelial cells to the modified LDL closely approximates linear behavior in scenario 1. Note that the capacity influx due to the response of the endothelial cells to the modified LDL was modeled to be proportional to the concentration modified LDL ($F_{m}l$) and will therefore always behave similarly. Moreover, the capacity concentration may exceed the limiting value for the modified LDL concentration of 0.3 mM, since capacity does not take up space until occupied by modified LDL.

### Model performance

**Fig. 4:**
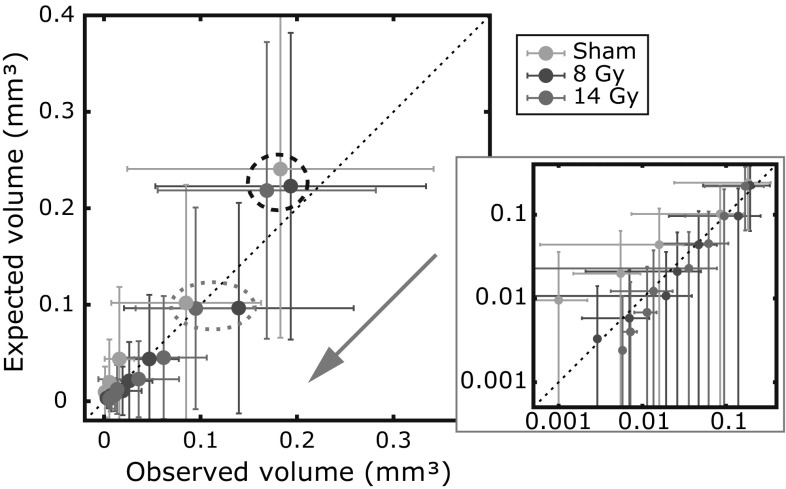
The expected modeled average plaque volumes are plotted versus the observed average plaque volumes for the sorted plaques for all three dose groups. The *error bars* show the standard deviation of the set of sorted plaque volumes, and indicate the range of corresponding volumes. The *gray arrow* indicates the initiation time after birth; the largest plaques were initiated in the beginning of a lifetime. The values corresponding to the first plaques ($q=1$) are encircled by the *black dashed ellipse* and the values corresponding to the second plaques ($q=2$) by the *gray dotted ellipse*. The same results are shown on a logarithmic scale on the *right* for an improved view for the smaller plaques. These results correspond to scenario 1, but similar results hold for scenario 2

The expected average plaque volumes based on the model results were compared to the observed average plaque volumes to visualize the performance of the proposed model. As described earlier, we consider sorted plaque volumes and the averages and corresponding standard deviations of both the observed and expected modeled volumes were determined for every dose group as described earlier. Since the results for both scenarios are very similar, Fig. 4 only shows the results from scenario 1. The values corresponding to the first plaques ($q=1$, i.e., oldest and largest plaques) in the three dose groups are encircled by the black dashed ellipse and the values corresponding to the second plaques ($q=2$) by the gray dotted ellipse. The direction of initiation is indicated by the gray arrow from oldest, and thus largest, to youngest and smallest plaques. Results are presented both on a linear and a logarithmic scale. As can be seen in Fig. 4, the standard deviations of observed and expected sorted volumes, represented by the error bars, are in the same order of magnitude as the corresponding average. The error bars give an indication of the range of volumes, and not for actual computation errors. The averages are well concentrated along the dashed line of equality $y=x$, indicating a strong agreement between the observed and expected volumes. Despite the limitations of the model and the limited data set of plaque sizes, the obtained results indicate a good model performance for all three dose groups.

## Discussion and conclusion

The model presented in this study was constructed to describe plaque development including possible radiation effects. This was accomplished by combining probabilistic dose-dependent plaque initiation and mechanistic modeling of biological processes underlying atherosclerosis that describes volumetric plaque growth. As a proof of concept, the model was tailored to experimental plaque size data from ApoE$^{{-/-}}$ mice to demonstrate how biologically-relevant information of the underlying mechanisms can easily be extracted. Since the experimental data set did not allow for pinpointing one unique scenario for atherosclerotic plaque development, we chose to show two typical scenarios to illustrate the applicability of the model. Plaque growth curves were comparable for both scenarios, but with different underlying mechanisms. Moreover, the plaque growth could not be described as linear with time. For these scenarios, the model performance was investigated by examining the relation between the observed average plaque volumes (sorted by initiation after birth) and the corresponding expected average plaque volumes based on the model outcome. Both scenarios showed similar results, for all three dose groups [0 (control), 8, and 14 Gy]: the expected average volumes were in good agreement with the observed average volumes with standard deviation in the same order as the average values. This indicates that this model approach may be well suitable to describe radiation-promoted atherosclerosis.

The model also has some restrictions and limitations. First of all, plaque growth was assumed to be identical for all plaques. This allows no individual variation in growth due to a plaque’s location, in this illustrative example: the position in the carotid artery. The model in its current state does also not contain dose-dependent growth. Although the authors are aware of the possible dose-related influences on the growth [e.g., the studies in Katayama et al. (2008); Hallahan et al. (1996)], the plaque size data showed no convincing dose-related growth. An explanation for the fact that the dose-related effects are not clearly recognizable can be the relatively short influence time of radiation of 14 days compared to the total growth time of a plaque. Another possible explanation can be the early age the mice were irradiated; most of the plaques had not been initiated at this age. Plaque size data from chronically-irradiated mice may be more useful to elucidate dose-related effects on plaque growth in the future.

Further, the governing equations were formulated to describe the early stages of plaque development, and do therefore not include for example the migration of smooth muscle cells and the growth of a necrotic core. Additionally the presence of high density lipoprotein (HDL), which is often assumed to reduce plaque development [e.g., the studies in Barter (2005); Joy and Hegele (2008); Williams et al. (2008)], was not included in the current model. In the current study, the plaque development process could thus be considered as a net initiation. Moreover, since radiation exposure is not likely to affect HDL levels (Tribble et al. 1999), this should not be a limitation when investigating dose-related effects.

Despite these limitations, this model is a first and promising attempt to describe radiation-promoted atherosclerosis based on experimental plaque size data. The search for the exact underlying biological mechanisms can be intensified with experimental data sets having a larger variety in experimental parameters, such as exposure profiles and maximum plaque growth times. Besides this, the model can be further developed by including additional radiation-related parameters or mechanisms. For example, an additional dose-related influx of modified LDL into the intima can be introduced. If this effect is sufficiently large, this can result in larger plaques for irradiated mice. However, such a modification will improve the model description, but not necessarily its usefulness since additional information on plaque composition or experimental values for parameters may be necessary for improved accuracy.

## Electronic supplementary material

Below is the link to the electronic supplementary material.

Supplement material (PDF 166 kb)
