# Supplementary material for: How radiation influences atherosclerotic plaque development: a biophysical approach in ApoE ¯/¯ mice
Source: Radiat Environ Biophys. 2017 Sep 2;56(4):423–31. doi: 10.1007/s00411-017-0709-2 (PMC5655690; doi:10.1007/s00411-017-0709-2)
Supplement: Supplementary file 1 — Supplement material (PDF 166 kb) [file 411_2017_709_MOESM1_ESM.pdf]

Supplementary material

Click here to view linked References

Click here to download attachment to manuscript Suppl_Mat_AKloosterman_TvDillen_ApoEmice.pdf

How radiation influences atherosclerotic plaque development: a biophysical approach in ApoE$^{-/-}$ mice – Supplementary material

Derivation of the modeled average plaque volume $\langle V_q^{\mathrm{mod}} \rangle$

The derivation of equation [7] for the modeled average plaque volume in the manuscript 'How radiation influences atherosclerotic plaque development: a biophysical approach in ApoE$^{-/-}$ mice' takes place in four distinct steps as outlined below.

Step-1: We first have to derive the average size of the qth plaque for a mouse having exactly n plaques at attained age T, with $q = 1, 2, \ldots, n$ (mouse index j is omitted for now). To this end, we define a conditional probability density function $f_{q,n}(t|T)$ for initiation events, such that $f_{q,n}(t|T)\Delta t$ is the probability that plaque number q is initiated between t and $t + \Delta t$, given the fact that there are n plaques at attained age T. This function is evaluated as:

$$f_{q,n}(t|T) = \lim_{\Delta t \to 0} \frac{\Pr\left(\text{plaque } q \text{ between } t \text{ and } t{+}\Delta t \middle| n \text{ plaques in } T\right)}{\Delta t}. \qquad \text{[S.1]}$$

The qth plaque being initiated between t and $t + \Delta t$ implies that $q-1$ plaques must have been initiated between 0 and t and $n-q$ plaques between $t+\Delta t$ and T, which has a probability:

$$\frac{\left(\Lambda_{0,t}\right)^{q-1}}{(q-1)!}\exp\left[-\Lambda_{0,t}\right]\frac{\left(\Lambda_{t,t+\Delta t}\right)^{1}}{1!}\exp\left[-\Lambda_{t,t+\Delta t}\right]\frac{\left(\Lambda_{t+\Delta t,T}\right)^{n-q}}{(n-q)!}\exp\left[-\Lambda_{t+\Delta t,T}\right]$$
$$= \frac{1}{(q-1)!(n-q)!}\Lambda_{t,t+\Delta t}\left(\Lambda_{0,t}\right)^{q-1}\left(\Lambda_{t+\Delta t,T}\right)^{n-q}\exp\left[-\Lambda_{0,T}\right].$$

Using $\Pr\left(n \text{ plaques in } T\right) = \Lambda_{0,T}^{n}\exp\left[-\Lambda_{0,T}\right]/n!$ and Bayes' theorem, equation [S.1] can be worked out as:

$$f_{q,n}(t|T) = \lim_{\Delta t \to 0}\frac{1}{\Delta t}\frac{\dfrac{1}{(q-1)!(n-q)!}\Lambda_{t,t+\Delta t}\left(\Lambda_{0,t}\right)^{q-1}\left(\Lambda_{t+\Delta t,T}\right)^{n-q}\exp\left[-\Lambda_{0,T}\right]}{\dfrac{\left(\Lambda_{0,T}\right)^{n}\exp\left[-\Lambda_{0,T}\right]}{n!}}$$

$$= \frac{n!}{(q-1)!(n-q)!}\frac{\left(\Lambda_{0,t}\right)^{q-1}}{\left(\Lambda_{0,T}\right)^{n}}\lim_{\Delta t \to 0}\frac{\Lambda_{t,t+\Delta t}\left(\Lambda_{t+\Delta t,T}\right)^{n-q}}{\Delta t},$$

which results in:

$$f_{q,n}\left(t\middle|T\right)=q\binom{n}{q}\lambda(t)\frac{\left(\Lambda_{0,t}\right)^{q-1}\left(\Lambda_{t,T}\right)^{n-q}}{\left(\Lambda_{0,T}\right)^{n}}. \qquad [S.2]$$

Equation [S.2] is a normalized probability density function, which means that

$$\int_0^T f_{q,n}\left(t\middle|T\right)\mathrm{d}t=1, \qquad [S.3]$$

for each $q\in\{1,2,...,n\}$ and each $n\in\{1,2,3,....\}$. This will be shown at the end of this document. Figure 1 shows an example of all ten distribution functions $f_{q,10}\left(t\middle|T\right)$ with $T=10$, $n=10$, $q\in\{1,2,...,10\}$, and with $\lambda(t)=\lambda_0=1$.

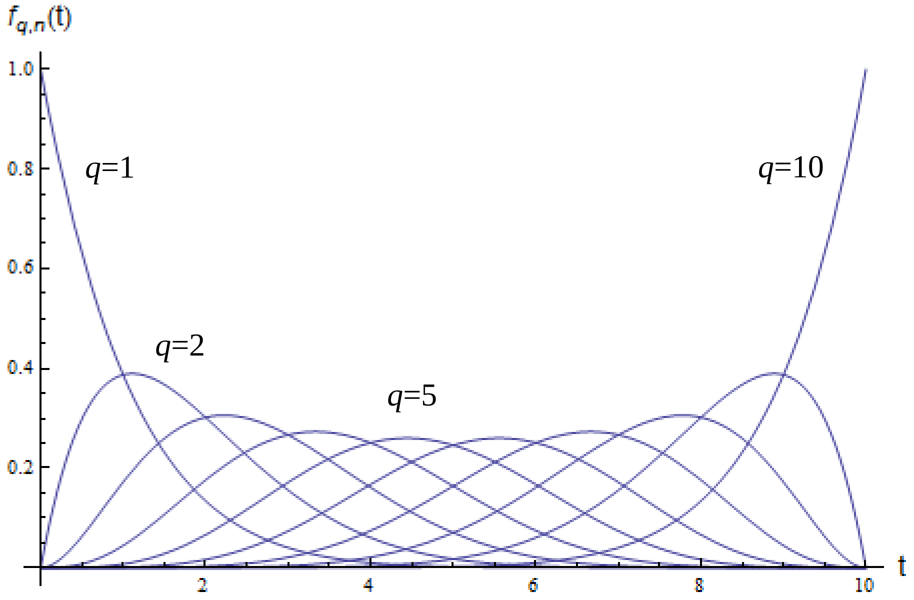

Figure 1: Ten conditional probability density function $f_{q,n}\left(t\middle|T\right)$ are shown for $q\in\{1,2,...,10\}$, with $n=10$, $T=10$, and $\lambda=\lambda_0=1$.

Using equation [S.2], the average volume of the qth plaque under the condition of n plaques at attained age T is:

$$\left\langle V_{q,n}^{\text{mod}}\right\rangle=\int_0^T f_{q,n}\left(t\middle|T\right)V\left(T-t\right)\mathrm{d}t, \qquad [S.4]$$

in which $T-t$ is the growth time of the plaque. The integrand in equation [S.4] is the conditional probability (density) of initiation time t (and thus the conditional probability of growth time $T-t$) multiplied by the corresponding plaque's volume at attained age T. The average volume is found by integrating over all possible initiation times between 0 and T.

Step-2: Within each dose group, each study subject may have a different exposure profile and/or attained age. Therefore, we need to distinguish between the subjects and reintroduce index j:

$$\left\langle V_{q,n_j}^{j,\mathrm{mod}} \right\rangle = \int_0^{T_j} f_{q,n_j}^{j}\left(t \middle| T_j\right) V_j\left(T_j - t\right) \mathrm{d}t \,, \tag{S.5}$$

with $V_j\left(T_j - t\right)$ the plaque's growth function of subject j with attained age T_j.

Step-3: The fact that subject j has n_j observed plaques is merely one realization (one specific drawing) under the Poisson process. Another (virtual) subject with the exact same exposure profile and attained age could just as well have had $n_j \pm 1$, $n_j \pm 2$, $n_j \pm 3$,... plaques (with different probabilities of course). In other words, we need to average over all possible realizations (drawings) of the Poisson distribution:

$$\left\langle V_{q}^{j,\mathrm{mod}} \right\rangle = \sum_{k=q}^{\infty} \Pr\left(k \text{ plaques in } T_j \middle| k \geq q\right) \left\langle V_{q,k}^{j,\mathrm{mod}} \right\rangle \,, \tag{S.6}$$

where n_j has been replaced by dummy index k. In equation [S.6] the conditional probability $\Pr\left(k \text{ plaques in } T_j \middle| k \geq q\right)$ is the Poisson probability of encountering a total of k plaques, of course under the condition of having more than q plaques (i.e. $k \geq q$). This probability can be worked out as follows:

$$\Pr\left(k \text{ plaques in } T_j \middle| k \geq q \right) = \frac{\Pr\left(k \text{ plaques in } T_j \cap k \geq q\right)}{\Pr\left(k \geq q \text{ plaques in } T_j\right)} \,. \tag{S.7}$$

Since the value of k is already equal or larger than q by construction of equation [S.6], the joint probability in the nominator of equation [S.7] is the Poisson probability of k plaques in T_j: $\Pr\left(k \text{ plaques in } T_j \cap k \geq q\right) = \Pr\left(k \text{ plaques in } T_j\right)$. With this information, equation [S.7] is worked out as:

$$\Pr\left(k \text{ plaques in } T_j \,\middle|\, k \geq q\right) = \frac{\Pr\left(k \text{ plaques in } T_j\right)}{\Pr\left(k \geq q \text{ plaques in } T_j\right)} = \frac{\left(\Lambda_{0,T_j}^{j}\right)^k \exp\left[-\Lambda_{0,T_j}^{j}\right] \big/ k!}{\sum_{r=q}^{\infty}\left(\left(\Lambda_{0,T_j}^{j}\right)^r \exp\left[-\Lambda_{0,T_j}^{j}\right] \big/ r!\right)}$$

$$= \frac{\left(\Lambda_{0,T_j}^{j}\right)^k \big/ k!}{\sum_{r=q}^{\infty}\left(\left(\Lambda_{0,T_j}^{j}\right)^r \big/ r!\right)} \equiv \frac{1}{Z_q^j}\frac{\left(\Lambda_{0,T_j}^{j}\right)^k}{k!}.$$

Thus, we have:

$$\Pr\left(k \text{ plaques in } T_j \,\middle|\, k \geq q\right) = \frac{1}{Z_q^j}\frac{\left(\Lambda_{0,T_j}^{j}\right)^k}{k!}, \qquad \text{[S.8]}$$

with normalization constant:

$$Z_q^j = \sum_{r=q}^{\infty}\frac{\left(\Lambda_{0,T_j}^{j}\right)^r}{r!}. \qquad \text{[S.9]}$$

Note that, by construction, the following required condition holds:

$$\sum_{k=q}^{\infty}\Pr\left(k \text{ plaques in } T_j \,\middle|\, k \geq q\right) = \sum_{k=q}^{\infty}\frac{1}{Z_q^j}\frac{\left(\Lambda_{0,T_j}^{j}\right)^k}{k!} = \frac{1}{Z_q^j}\sum_{k=q}^{\infty}\frac{\left(\Lambda_{0,T_j}^{j}\right)^k}{k!} = \frac{1}{Z_q^j}Z_q^j = 1.$$

Inserting equations [S.8], [S.5] (with n_j replaced by k) and [S.2] into equation [S.6] yields:

$$\left\langle V_q^{j,\text{mod}}\right\rangle = \sum_{k=q}^{\infty}\Pr\left(k \text{ plaques in } T_j \,\middle|\, k \geq q\right)\left\langle V_{q,k}^{j,\text{mod}}\right\rangle = \sum_{k=q}^{\infty}\frac{1}{Z_q^j}\frac{\left(\Lambda_{0,T_j}^{j}\right)^k}{k!}\int_0^{T_j} f_{q,k}^{j}\left(t \,\middle|\, T_j\right)V_j\left(T_j - t\right)\mathrm{d}t$$

$$= \sum_{k=q}^{\infty}\frac{1}{Z_q^j}\frac{\left(\Lambda_{0,T_j}^{j}\right)^k}{k!}\int_0^{T_j} q\binom{k}{q}\lambda_j(t)\frac{\left(\Lambda_{0,t}^{j}\right)^{q-1}\left(\Lambda_{t,T_j}^{j}\right)^{k-q}}{\left(\Lambda_{0,T_j}^{j}\right)^k}V_j\left(T_j - t\right)\mathrm{d}t$$

$$= \frac{1}{Z_q^j}\sum_{k=q}^{\infty}\frac{\left(\Lambda_{0,T_j}^{j}\right)^k}{\left(\Lambda_{0,T_j}^{j}\right)^k}\frac{q}{k!}\binom{k}{q}\int_0^{T_j}\lambda_j(t)\left(\Lambda_{0,t}^{j}\right)^{q-1}\left(\Lambda_{t,T_j}^{j}\right)^{k-q}V_j\left(T_j - t\right)\mathrm{d}t$$

$$= \frac{q}{Z_q^j}\sum_{k=q}^{\infty}\frac{1}{k!}\binom{k}{q}\int_0^{T_j}\lambda_j(t)\left(\Lambda_{0,t}^{j}\right)^{q-1}\left(\Lambda_{t,T_j}^{j}\right)^{k-q}V_j\left(T_j - t\right)\mathrm{d}t.$$

We thus have the following result:

$$\left\langle V_q^{j,\text{mod}} \right\rangle = \frac{q}{Z_q^j} \sum_{k=q}^{\infty} \frac{1}{k!} \binom{k}{q} \int_0^{T_j} \lambda_j(t) \left(\Lambda_{0,t}^j\right)^{q-1} \left(\Lambda_{t,T_j}^j\right)^{k-q} V_j\left(T_j - t\right) \mathrm{d}t \,. \qquad \text{[S.10]}$$

Step-4: Finally, we have to average over all N subjects in the dose group we are studying:

$$\left\langle V_q^{\text{mod}} \right\rangle = \frac{1}{N} \sum_{j=1}^{N} \left\langle V_q^{j,\text{mod}} \right\rangle . \qquad \text{[S.11]}$$

Inserting equation [S.10] into [S.11] then yields our final result:

$$\left\langle V_q^{\text{mod}} \right\rangle = \frac{1}{N} \sum_{j=1}^{N} q \left(Z_q^j\right)^{-1} \sum_{k=q}^{\infty} \frac{1}{k!} \binom{k}{q} \int_0^{T_j} \lambda_j(t) \left(\Lambda_{0,t}^j\right)^{q-1} \left(\Lambda_{t,T_j}^j\right)^{k-q} V_j\left(T_j - t\right) \mathrm{d}t \,. \qquad \text{[S.12]}$$

Equation [7] is equal to equation [S.12], with all time-related functions denoted with a hat to indicate that these functions are evaluated using the optimal set of parameters $\left\{\hat{\boldsymbol{\theta}}, \hat{\boldsymbol{\beta}}\right\} = \arg\max_{\boldsymbol{\theta},\boldsymbol{\beta}} Dev\left(\boldsymbol{\theta}, \boldsymbol{\beta}\right)$ found by the maximum-likelihood fitting routine described in the manuscript.

Proof of normalization condition [S.3]

We will prove that the normalization condition of the conditional probability density function $f_{q,n}\left(t\middle|T\right)$ holds:

$$\int_0^T f_{q,n}\left(t\middle|T\right)\mathrm{d}t = 1, \tag{S.3}$$

We start by rewriting $f_{q,n}\left(t\middle|T\right)$ as follows:

$$f_{q,n}\left(t\middle|T\right) = q\binom{n}{q} g(t)\left(G(t)\right)^{q-1}\left(1-G(t)\right)^{n-q}, \tag{S.13}$$

with

$$g(t) = \frac{\lambda(t)}{\Lambda_{0,T}}, \tag{S.14}$$

and its primitive counterpart

$$G(t) = \frac{\Lambda_{0,t}}{\Lambda_{0,T}}. \tag{S.15}$$

Note that by the definition of equation [S.15], we can simply rewrite $\frac{\Lambda_{t,T}}{\Lambda_{0,T}}$ as $\frac{\Lambda_{0,T}-\Lambda_{0,t}}{\Lambda_{0,T}} = 1-G(t)$, which has been used in equation [S.13] as well. Furthermore, we know that $G(0)=0$ and $G(T)=1$. So, we are facing the following integral for normalization:

$$I = \int_0^T f_{q,n}\left(t\middle|T\right)\mathrm{d}t = q\binom{n}{q}\int_0^T g(t)\left(G(t)\right)^{q-1}\left(1-G(t)\right)^{n-q}\,\mathrm{d}t\,. \tag{S.16}$$

We apply the method of substitution of variables to rewrite this integral:

$$\begin{aligned}\int_0^T g(t)\left(G(t)\right)^{q-1}\left(1-G(t)\right)^{n-q}\,\mathrm{d}t &= \int_{G(0)}^{G(T)}\left(G(t)\right)^{q-1}\left(1-G(t)\right)^{n-q}\,\mathrm{d}G(t)\\ &= \int_0^1 x^{q-1}\left(1-x\right)^{n-q}\,\mathrm{d}x.\end{aligned}$$

Thus we can write:

$$I = q\binom{n}{q}\int_0^1 x^{q-1}(1-x)^{n-q}\,\mathrm{d}x\,. \qquad [S.17]$$

Using the beta function $B(a,b)=\int_0^1 x^{a-1}(1-x)^{b-1}\,\mathrm{d}x$ with $a=q$ and $b=n-q+1$, we can write equation [S.17] as:

$$I = q\binom{n}{q}B(q,n-q+1)\,. \qquad [S.18]$$

With the property $B(a,b)=\frac{(a-1)!(b-1)!}{(a+b-1)!}$ for positive integers *a* and *b*, equation [S.18] is then finally worked out as:

$$I = q\binom{n}{q}\frac{(q-1)!(n-q+1-1)!}{(q+n-q+1-1)!} = \frac{n!}{(q-1)!(n-q)!}\frac{(q-1)!(n-q)!}{n!} = 1\,. \qquad [S.19]$$
